# Real‐world data of atezolizumab plus carboplatin and etoposide in elderly patients with extensive‐disease small‐cell lung cancer

**DOI:** 10.1002/cam4.4938

**Published:** 2022-06-14

**Authors:** Ayako Shiono, Hisao Imai, Satoshi Wasamoto, Takeshi Tsuda, Yoshiaki Nagai, Hiroyuki Minemura, Yutaka Yamada, Takayuki Kishikawa, Yukihiro Umeda, Hiroki Takechi, Ou Yamaguchi, Atsuto Mouri, Kyoichi Kaira, Hirokazu Taniguchi, Koichi Minato, Hiroshi Kagamu

**Affiliations:** ^1^ Department of Respiratory Medicine, International Medical Center Saitama Medical University Hidaka Japan; ^2^ Division of Respiratory Medicine Gunma Prefectural Cancer Center Ota Japan; ^3^ Division of Respiratory Medicine Saku Central Hospital Advanced Care Center Saku Japan; ^4^ Division of Respiratory Medicine Toyama Prefectural Central Hospital Toyama Japan; ^5^ Department of Respiratory Medicine Jichi Medical University, Saitama Medical Center Saitama Japan; ^6^ Department of Pulmonary Medicine Fukushima Medical University Fukushima Japan; ^7^ Division of Respiratory Medicine Ibaraki Prefectural Central Hospital Kasama Japan; ^8^ Division of Thoracic Oncology Tochigi Cancer Center Utsunomiya Japan; ^9^ Third Department of Internal Medicine, Faculty of Medical Sciences University of Fukui Eiheiji Japan

**Keywords:** atezolizumab plus carboplatin and etoposide, elderly patients, immune checkpoint inhibitor, small‐cell lung cancer

## Abstract

**Purpose:**

The aim of this study was to assess the effectiveness and tolerability of atezolizumab plus carboplatin and etoposide combination chemotherapy in elderly patients with extensive‐disease (ED) small‐cell lung cancer (SCLC).

**Methods:**

This retrospective study evaluated 65 SCLC patients who received atezolizumab, carboplatin, and etoposide for ED‐SCLC in nine study institutions between August 2019 and September 2020. Clinical efficacy, assessed according to response rate and survival, and toxicity were compared between the elderly (*n* = 36 patients; median age: 74 years [range: 70–89 years]) and the non‐elderly group (*n* = 29 patients; median age: 67 years [range: 43–69 years]).

**Results:**

The response rate was 73.8% (80.5% in the elderly group and 65.5% in the non‐elderly group). There was no significant difference in both the median progression‐free survival (5.5 months vs. 4.9 months, *p* = 0.18) and the median overall survival (15.4 months vs. 15.9 months, *p* = 0.24) between the elderly group and the non‐elderly group. The frequencies of grade ≥3 hematological adverse events in the elderly patients were as follows: decreased white blood cells, 36.1%; decreased neutrophil count, 61.1%; decreased platelet count, 8.3%; and febrile neutropenia, 8.3%. One treatment‐related death due to lung infection occurred in the elderly group.

**Conclusion:**

Despite hematologic toxicities, especially decreased neutrophil count, atezolizumab, carboplatin, and etoposide combination chemotherapy demonstrates favorable effectiveness and acceptable toxicity in elderly patients. Thus, atezolizumab plus carboplatin and etoposide could be the preferred standard treatment modality for elderly patients with ED‐SCLC.

## Graphical abstract

The aim of this study was to assess the effectiveness and tolerability of atezolizumab plus carboplatin and etoposide combination chemotherapy in elderly patients with extensive‐disease (ED) small‐cell lung cancer (SCLC). There was no significant difference in both the median progression‐free survival (5.5 months vs 4.9 months, p=0.18) and the median overall survival (15.4 months vs 15.9 months, p=0.24) between the elderly group and the non‐elderly group. Atezolizumab plus carboplatin and etoposide could be the preferred standard treatment modality for elderly patients with ED‐SCLC.

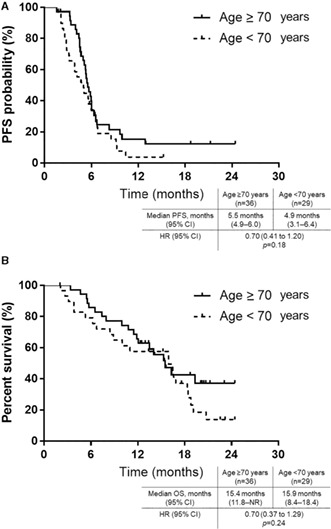

## 1 INTRODUCTION

Small‐cell lung cancer (SCLC) is characterized by exponential progression and metastasis, and is reported to account for 10%–15% of all lung cancers.^1^ Nearly two‐thirds of SCLC cases have extensive disease (ED) at diagnosis, which is correlated with poor prognosis.^2^ Chemotherapy can relieve symptoms and prolong survival in most ED‐SCLC patients, but long‐term period survival is unusual.^3^, ^4^ Until a few years ago, the standard first‐line therapy for patients with ED‐SCLC was chemotherapy with platinum and etoposide. The median survival time was roughly 10 months, and no significant improvement in overall survival (OS) had been reported for more than 20 years.^5^, ^6^ Before the introduction of immune checkpoint inhibitors (ICIs), ED‐SCLC was a malignancy with a reported first‐line objective response rate (ORR) of 44%–78%, median progression‐free survival (PFS) of 4.3–5.7 months, median OS of 7.5–10.9 months, and a 5‐year survival rate of just 2.8%.^6^, ^7^ However, ICIs have recently shown improved survival in patients with ED‐SCLC.^8^, ^9^, ^10^

The occurrence of thoracic malignancy rises with age. Accordingly, the incidence is also currently increasing in parallel with the gain in life expectancy worldwide. A disproportionate impact on the elderly population has been observed, along with a significant gain in the occurrence of lung malignancy in the older adult. More than 50% of lung cancer patients are diagnosed at an age of 65 or older, the lower limit of what is defined as “elderly” in epidemiological studies.^11^ Further, about 30%–40% of SCLC patients are reported to be over 70 years old at the time of diagnostication.^12^ Thus, the strategy for individualizing drug therapy in older patients with SCLC is a relevant clinical problem.

To date, four cycles of carboplatin plus etoposide remain the standard first‐line therapeutic regimen for older adults patients with ED‐SCLC.^13^ Elderly patients are less tolerant to chemotherapy and require medical management based on individual‐level parameters such as metastatic site, general condition, laboratory data, quality of life, self‐management ability, and organ function, further complicating individual patient care.^14^, ^15^ It is still unclear whether standard chemotherapy for the elderly can be used safely in clinical practice.^16^ Although the median age of lung cancer patients at the time of diagnostication is reported to be 70 years, just a small proportion of older adults patients are enrolled in clinical trials.^17^

Atezolizumab is a humanized monoclonal anti‐programmed death ligand 1 (PD‐L1) antibody, an immune checkpoint inhibitor that blocks PD‐L1‐programmed death 1 (PD‐1) and PD‐L1‐B7‐1 signaling and recovers tumor‐specific T‐cell immunity.^18^ The landmark IMpower133 study compared the addition of atezolizumab to carboplatin and etoposide with the addition of placebo to carboplatin and etoposide in the first‐line therapy of patients with ED‐SCLC. The median PFS period and median OS period were significantly better in patients who received atezolizumab plus carboplatin and etoposide than in patients who received placebo plus carboplatin and etoposide (5.2 months vs. 4.3 months and 12.3 months vs. 10.3 months, respectively).^9^, ^19^ Regarding safety, it was reported that there was no significant difference in the signal of treatment‐related toxicities between the two cohorts Meanwhile, treatment‐related quality of life was better in patients who received atezolizumab plus carboplatin and etoposide than in patients who received placebo plus carboplatin and etoposide.^20^ Moreover, durvalumab, another PD‐L1 antibody, was also revealed to have a comparable treatment efficacy in the CASPIAN study (median OS = 12.9 months vs. 10.5 months).^10^, ^21^ Pembrolizumab, a PD‐1 antibody, was also studied in the KEYNOTE‐604 study; although it improved the PFS, there was no significant difference in OS.^22^

Although systemic treatment consisting of ICIs plus platinum and etoposide combination therapy is considered to be an effective treatment for ED‐SCLC based on the results of the aforementioned studies, the effectiveness and feasibility of atezolizumab, carboplatin, and etoposide in older adult patients with ED‐SCLC have not been fully assessed. Thus, this current study aimed to evaluate the activity and feasibility of atezolizumab plus carboplatin and etoposide among elderly patients with ED‐SCLC.

## 2 METHODS

### 2.1 Study patients

This study analyzed the records of patients diagnosed with ED‐SCLC who received the therapeutic regimen of atezolizumab plus carboplatin and etoposide between August 2019 and September 2020 in nine Japanese institutions. The design of this study is retrospective. The inclusion criteria were (1) cytological or histological diagnosis of SCLC with stage III/IV with no curative irradiation or postoperative recurrence and (2) first‐line chemotherapeutic regimen with atezolizumab, carboplatin, and etoposide combination chemotherapy. The elderly was defined as patients aged ≥70 years; patients aged <70 years were categorized as non‐elderly. Pathological stage III/IV SCLC was determined based on the Union for International Cancer Control tumor–node–metastasis (TNM) Classification, Seventh Edition.

All patients underwent pretreatment physical exam, chest radiography, thoracoabdominal computed tomography, brain computed tomography or magnetic resonance imaging, and^18^F‐fluorodeoxyglucose positron emission tomography or bone scintigraphy to assess the disease stage of TNM. The electronic medical charts of the patients to be studied were searched and the data of each patient were collected.

### 2.2 Treatment and response evaluation

All the patients had no history of atezolizumab, carboplatin, and etoposide combination therapy, and each patient was administered up to four cycles of atezolizumab (fixed dose of 1200 mg, intravenous injection on day 1 of each cycle), carboplatin (area under the curve of 4–5 min mg/ml, intravenous injection on day 1 of each cycle), and etoposide (80–100 mg/m^2^ body surface area, intravenous injection on days 1 through 3 of each cycle), followed by atezolizumab maintenance every 3 weeks. Granulocyte colony‐stimulating factor was administered as prophylaxis against neutropenia at the discretion of the attending physician. Treatment was terminated when disease progression was observed, intolerable toxicity occurred, or the patient withdrew consent for treatment.

Radiographic treatment responses were evaluated according to the best overall treatment response and maximum tumor shrinkage based on the Response Evaluation Criteria in Solid Tumors, version 1.1.^23^ Tumor responses were classified as complete response (CR), partial response (PR), stable disease (SD), progressive disease (PD), and not evaluated (NE). If treatment failure occurred, patients were permitted any subsequent therapy of their preference, including continuation of atezolizumab maintenance therapy. Treatment toxicities associated with the combination chemotherapy were graded following the Common Terminology Criteria for Adverse Events (version 5.0).

### 2.3 Statistical analysis

Categorical variables were calculated using Fisher's exact test, and continuous variables were calculated using Welch's *t*‐test. PFS was calculated from the first day of treatment until progressive disease (PD) or death from any reason. OS was calculated from the initiation of treatment until death or was censored on the day of the last visit. PFS and OS were analyzed with the Kaplan–Meier method and compared between the two groups using the log‐rank test. Differences were considered statistically significant at a two‐tailed *p*‐value of <0.05. All statistical analyses were performed using the JMP statistical software, version 11.0, for Windows (SAS Institute, Cary, NC).

## 3 RESULTS

### 3.1 Patient characteristics

**FIGURE 1:**
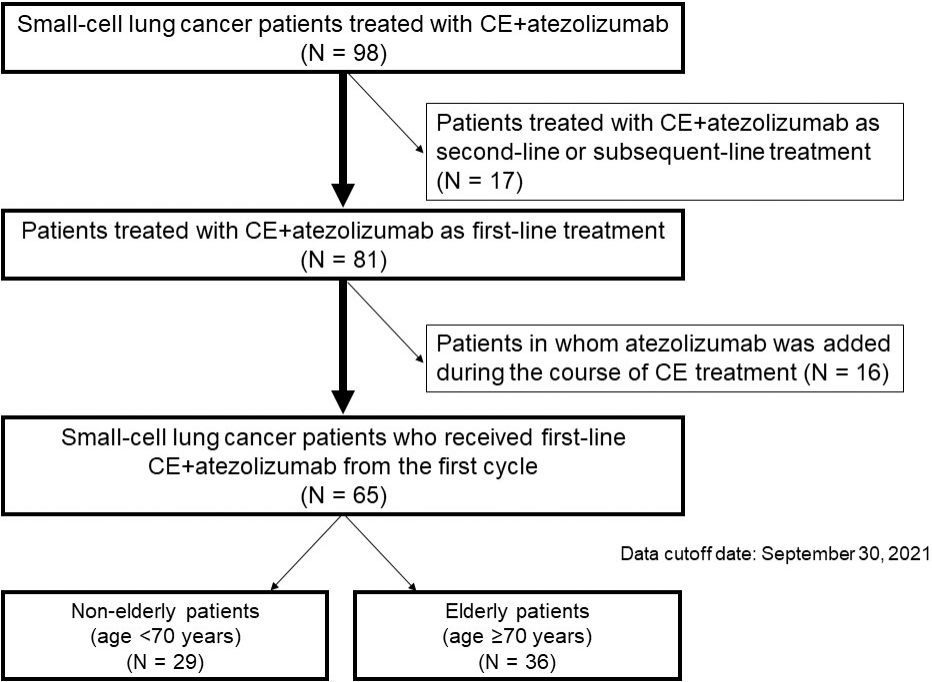
Patient selection diagram. Patients treated with atezolizumab plus CE between August 2019 and September 2020 were included. CE, carboplatin and etoposide.

A total of 98 patients were administered combination chemotherapy with atezolizumab plus carboplatin and etoposide. Among them, chemotherapy was performed as second‐ or subsequent‐line treatment in 17 patients, and atezolizumab was added during the chemotherapy in 16 patients. To maintain uniformity in patient background, 33 patients were excluded. Finally, 65 patients were evaluated; of them, 36 and 29 patients were aged ≥70 and < 70 years, respectively. The patient selection diagram is shown in Figure 1.

Table 1 shows the patient characteristics by age group. Except for age, there were no statistically significant differences in patient background between the elderly and non‐elderly groups. The median number of atezolizumab plus carboplatin and etoposide administration cycles was four (range 1–4) in the non‐elderly cohort and four (range 2–4) in the elderly cohort. With respect to the doses of carboplatin and etoposide, the most common doses in the non‐elderly group were AUC 5 for carboplatin and 100 mg/m^2^ for etoposide (*n* = 27 patients, 93.1%). The other doses were used in only two patients. In the elderly group, the most common doses were also AUC 5 for carboplatin and 100 mg/m^2^ for etoposide (*n* = 23 patients, 63.8%). Dose reductions for etoposide and for both carboplatin and etoposide were made in 6 and 7 patients, respectively.

**TABLE 1:** Baseline patient characteristics

Characteristic	Total patients (*n* = 65)	Non‐elderly patients (age < 70 years) (*n* = 29)	Elderly patients (age ≥ 70 years) (*n* = 36)	*p*‐value^a^
Sex				
Male/female	54/11	24/5	30/6	>0.99
Age (years)				
Median	70	67	74	–
Range	43–89	43–69	70–89	
PS				
0/1/2/3/4	13/45/5/2/0	4/21/4/0/0	9/24/1/2/0	
Smoking status				
Yes/no	62/3	29/0	33/3	0.24
Histology				
Small‐cell carcinoma/combined small‐cell carcinoma	63/2	28/1	35/1	>0.99
Disease stage				
3/4/recurrence	5/57/3	1/27/1	4/30/2	
History of postoperative adjuvant chemotherapy				
Yes/no	1/64	0/29	1/35	>0.99
Intracranial metastases				
Yes/no	19/46	10/19	9/27	0.42
Liver metastases				
Yes/no	14/51	5/24	9/27	0.55
Bone metastases				
Yes/no	22/43	8/21	14/22	0.43
Number of administered cycles of carboplatin + etoposide + atezolizumab				
Median	4	4	4	0.32^b^
Range	1–4	1–4	2–4	
Number of administered cycles of atezolizumab maintenance therapy				
Median	2	2	2	0.81^b^
Range	0–24	0–15	0–24	
Starting dose				
CBDCA (AUC 5) + etoposide (100 mg/m^2^)	50	27	23	
CBDCA (AUC 5) + etoposide (80–99 mg/m^2^)	6	0	6	
CBDCA (AUC 4) + etoposide (100 mg/m^2^)	0	0	0	
CBDCA (AUC 4) + etoposide (80–99 mg/m^2^)	8	1	7	
CBDCA (AUC 5) + etoposide (<80 mg/m^2^)	1	1	0	
With granulocyte colony‐stimulating factor prophylaxis				
Yes/no	33/32	16/13	17/19	0.62
Prior radiotherapy				
Yes/no	7/58	2/27	5/31	0.44
Reason for discontinuation of carboplatin + etoposide + atezolizumab^c^				
Progressive disease	7	6	1	
Adverse events	3	2	1	
Others	5	2	3	
Steroid treatment for adverse events^d^				
Yes/no	6/59	3/26	3/33	>0.99
Continuing administration of atezolizumab at data cutoff	5/60	1/28	4/32	0.37

Abbreviations: AUC, area under the curve; CBDCA, Carboplatin; PS, Performance Status.

^a^ Comparison between elderly patients and non‐elderly patients.

^b^ Welch's *t*‐test.

^c^ Excluding atezolizumab maintenance therapy.

^d^ Excluding topical agents.

### 3.2 Treatment response

In the overall cohort, the overall response rate (ORR) was 73.8% (95% CI: 61.9–83.0), and the disease control rate was 89.2% (95% CI: 79.1–94.9). Treatment response according to the patient group is shown in Table 2. In total, 3, 16, and 6 patients in the non‐elderly group achieved CR, PR, and SD, respectively, while four patients developed PD. The total ORR was 65.5% (95% CI: 47.2–80.1). Meanwhile, 2, 27, and 4 patients in the elderly group achieved CR, PR, and SD, respectively, while two patients developed PD, and one patient was NE. The total response rate was 80.5% (95% CI: 64.6–90.5). There were no significant between‐group differences in the response rate and disease control rate. Table S1 (online only) shows a comparison of treatment response between patients aged 70–74 and ≥ 75 years. Among the patients aged 70–74 years, 1, 20, 2, 0, and 1 patient achieved CR, PR, SD, PD, and NE, respectively, with a total response rate of 87.5% (95% CI: 68.1–96.4). Meanwhile, among patients aged ≥75 patients, 1, 7, 2, and 2 patients achieved CR, PR, SD, and PD, respectively, with a total response rate of 83.3% (95% CI: 53.9–96.5). Among the elderly patients, the response rate and disease control rate were also not significantly different between those aged 70–74 years and ≥ 75 years.

**TABLE 2:** Treatment response

	Total (*n* = 65)	Non‐elderly patients (age < 70 years) (*n* = 29)	Elderly patients (age ≥ 70 years) (*n* = 36)	*p*‐value^a^
Response				
Complete response	5	3	2	
Partial response	43	16	27	
Stable disease	10	6	4	
Progressive disease	6	4	2	
Not evaluated	1	0	1	
Response rate, % (95% CI)	73.8 (61.9–83.0)	65.5 (47.2–80.1)	80.5 (64.6–90.5)	0.25
Disease control rate, % (95% CI)	89.2 (79.1–94.9)	86.2 (68.2–95.1)	91.6 (77.4–97.8)	0.69

Abbreviation: CI, confidence interval.

^a^ Comparison between elderly patients and non‐elderly patients.

### 3.3 Survival

**FIGURE 2:**
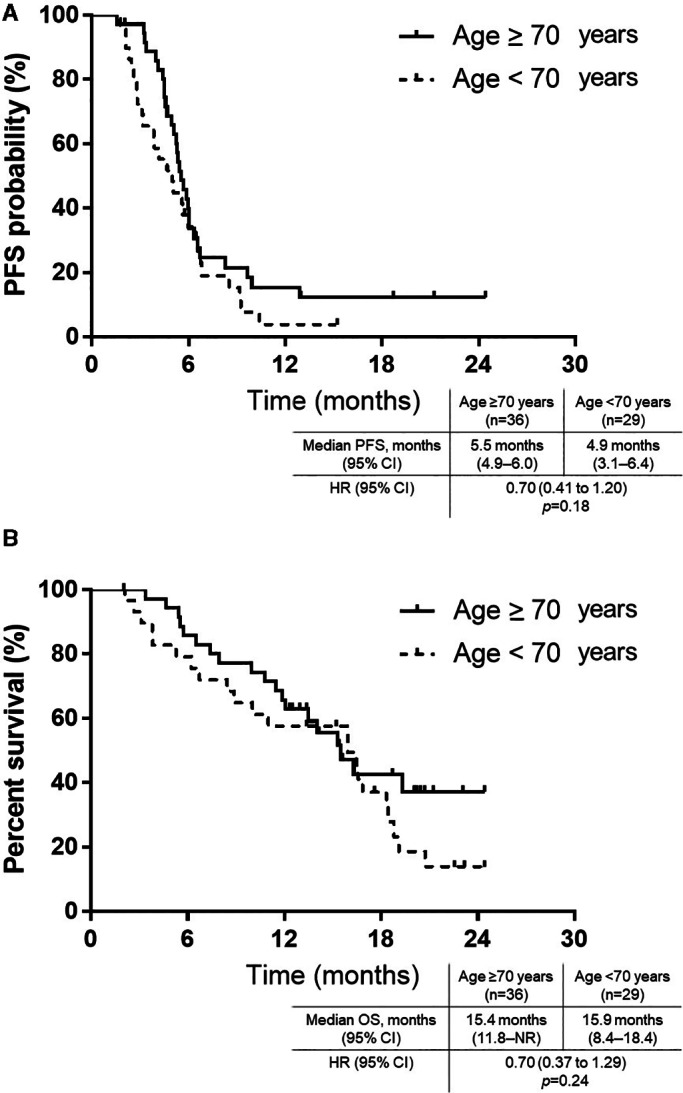
Kaplan–Meier curves of survival. (A) Progression‐free survival (PFS) of small‐cell lung cancer patients administered atezolizumab plus carboplatin and etoposide according to age: <70 years (dotted line) and ≥ 70 years (solid line). PFS does not significantly differ with age (median: 4.9 months for patients aged <70 years vs. 5.5 months for patients aged ≥70 years, *p* = 0.18). (B) Overall survival (OS) of small‐cell lung cancer patients administered atezolizumab plus carboplatin and etoposide according to age: <70 years (dotted line) and ≥ 70 years (solid line). OS does not significantly differ with age (median: 15.9 months for patients aged <70 years vs. 15.4 months for patients aged ≥70 years, *p* = 0.24).

The median follow‐up period in the overall population was 13.3 months (range 2.0–24.4 months). At that median follow‐up period, the median PFS and OS were 5.4 months (95% CI: 4.6–5.9 months) and 15.9 months (95% CI: 11.8–18.3 months), respectively. In total, 57 (87.6%) patients experienced disease progression and 41 (63.0%) patients died from events related to the primary disease. PFS was similar in both groups (Figure 2A). The median PFS was 4.9 months (95% CI: 3.1–6.4) in the non‐elderly cohort and 5.5 months (95% CI: 4.9–6.0) in the elderly cohort (log‐rank *p* = 0.18). The OS based on age (≥70 years/<70 years) is shown in Figure 2B. The median OS was 15.9 (95% CI: 8.4–18.4) months in the non‐elderly cohort and 15.4 (95% CI: 11.8–not reached) months in the elderly cohort (log‐rank *p* = 0.24). In addition, no significant difference was observed in any of the variables in the multivariate analysis of the entire patient population, except PFS according to prior radiotherapy (Table S2, online only).

In the elderly patients, the median PFS was 5.9 (95% CI: 5.2–8.2) months in the patients aged 70–74 years and 4.8 (95% CI: 3.2–6.0) months in those aged ≥75 years (log‐rank *p* = 0.09, Figure S1). Besides, the median OS was 14.0 (95% CI: 10.8–not reached) months in those aged 70–74 years and 16.3 months (95% CI: 5.4–not reached) in those aged ≥75 years (log‐rank *p* = 0.90, Figure S2). In addition, no significant difference in any of the variables was observed in the univariate analysis involving the elderly patients' data (Table S3, online only). Therefore, there were no significant differences in PFS and OS among patients aged 70–74 years and ≥ 75 years.

### 3.4 Toxicity

All 65 patients were assessed for treatment‐related adverse events. The toxicities are demonstrated in Table 3. The most common treatment‐related adverse event was myelosuppression. In the non‐elderly cohort, 27.5% of patients had a grade 3–4 decrease in white blood cells and 48.2% had a grade 3–4 decrease in neutrophil count. Meanwhile, in the elderly cohort, 36.1% of the patients had a grade 3–4 decrease in white blood cells, and 61.1% had a grade 3–4 decrease in neutrophil count. Febrile neutropenia was observed in 6.8% in the non‐elderly cohort and in 8.3% in the elderly cohort. The occurrence of immune‐related adverse events was low. In the elderly patients, the grade 3–4 immune‐related adverse events were skin rash, pneumonitis, adrenal insufficiency, myositis, and increased creatine phosphokinase (2.7% each). In the elderly group, one patient each developed grade 2 and grade 3 pneumonia. One patient had treatment‐related death. This patient had bacterial lung infection.

**TABLE 3:** Adverse events

	All patients (*n* = 65)				Non‐elderly patients (age < 70 years) (*n* = 29)				Elderly patients (age ≥ 70 years) (*n* = 36)			
Adverse event	Any grade	%	Grade ≥3	%	Any grade	%	Grade ≥3	%	Any grade	%	Grade ≥3	%
Led to discontinuation	6		5		4	13.7	3	10.3	2	5.5	2	5.5
Led to death	–		1		–	–	0	0	–	–	1	2.7
Treatment related^a^												
White blood cell count decrease	42	64.6	21	32.3	18	62	8	27.5	24	66.6	13	36.1
Neutrophil count decrease	43	66.1	36	55.3	19	65.5	14	48.2	24	66.6	22	61.1
Anemia	35	53.8	1	1.5	15	51.7	0	0	20	55.5	1	2.7
Platelet count decrease	33	50.7	4	6.1	15	51.7	1	3.4	18	50	3	8.3
Febrile neutropenia	5	7.6	5	7.6	2	6.8	2	6.8	3	8.3	3	8.3
Nausea	16	24.6	1	1.5	8	27.5	0	0	8	8.3	1	2.7
Anorexia	19	29.2	1	1.5	11	37.9	0	0	8	8.3	1	2.7
Creatinine increase	4	6.1	1	1.5	2	6.8	0	0	2	5.5	1	2.7
ALT increase	13	20	2	3	8	27.5	2	6.8	5	13.8	0	0
Infection	5	7.6	5	7.6	1	3.4	1	3.4	4	11.1	4	11.1
Hyponatremia	2	3	1	1.5	0	0	0	0	2	5.5	1	2.7
Thromboembolic event	1	1.5	1	1.5	0	0	0	0	1	2.7	1	2.7
Immune‐related^b^												
Rash	3	4.6	1	1.5	2	6.8	0	0	1	2.7	1	2.7
Hypothyroidism	6	9.2	1	1.5	5	17.2	1	3.4	1	2.7	0	0
Pneumonitis	2	3	1	1.5	0	0	0	0	2	5.5	1	2.7
Adrenal insufficiency	1	1.5	1	1.5	0	0	0	0	1	2.7	1	2.7
Myositis	1	1.5	1	1.5	0	0	0	0	1	2.7	1	2.7
CPK increased	1	1.5	1	1.5	0	0	0	0	1	2.7	1	2.7

Abbreviations: ALT, alanine aminotransferase; CPK, creatine phosphokinase.

^a^ Grade ≥3 treatment‐related adverse events reported in ≥1 patient.

^b^ Treatment‐related adverse events reported in ≥1 patient.

### 3.5 Subsequent treatments

The treatments administered following progressive disease are listed in Table 4. One patient was transferred to another doctor after atezolizumab plus carboplatin and etoposide treatment; thus, we could not obtain detailed information on the second‐ and subsequent‐line treatments. Among the 31 elderly patients who developed relapse, 23 patients received anticancer treatments. As an initial treatment following progressive disease, the most common second‐line treatment was amrubicin monotherapy, and the most common third‐ or subsequent‐line treatment was topotecan monotherapy. Eight patients were treated with best supportive care.

**TABLE 4:** Subsequent treatment of elderly patients

	Second‐line	Third‐line	≥Fourth‐line
Carboplatin + etoposide	2	1	1
Carboplatin + paclitaxel	0	0	1
Amrubicin	19	1	1
Topotecan	1	6	1
CPT‐11	1	1	1
Others	0	1	1
Best supportive care	8	‐	‐

## 4 DISCUSSION

The efficacy and feasibility of atezolizumab, carboplatin, and etoposide combination chemotherapy in elderly SCLC patients are yet to be clarified. In this study, atezolizumab, carboplatin, and etoposide combination chemotherapy showed favorable effectiveness and no new safety concerns in elderly patients, indicating its feasibility for older adult SCLC patients. In the overall population enrolled in this analysis, the treatment ORR, median PFS, and median OS were shown to be 73.8%, 5.4 months, and 15.9 months, respectively. These results are comparable or better than the overall response rate, median PFS, and median OS of 60.2%, 5.2 months, and 12.3 months, respectively, in the atezolizumab plus carboplatin and etoposide arm of the previous phase III IMpower133 trial.^9^ In addition, the tumor response was similar, while the median PFS and OS in the current study were better than those in the clinical practice data of atezolizumab plus carboplatin and etoposide in Korea, which reported the ORR, median PFS, and median OS of 75.0%, 4.6 months, and 12.0 months, respectively.^24^

As the population ages, the number of SCLC patients among the elderly is expected to increase.^25^, ^26^ Older adults with a good performance status (PS) and good organ function tend to receive the same chemotherapeutic regimens as those in younger adults. However, even older adults with good PS and good organ function have been reported to be at greater risk of serious toxicities than their younger counterparts.^27^, ^28^ The efficacy and safety of the combination regimen of carboplatin and etoposide have been reported in clinical trials for elderly patients with SCLC.^13^ Thus, based on the results of the clinical trial, carboplatin and etoposide combination chemotherapy without cisplatin has been used as the standard of care in clinical practice for elderly patients. Meanwhile, chemotherapeutic options for older adult patients with ED‐SCLC are still limited. Atezolizumab plus carboplatin and etoposide treatment has been widely used in recent years for elderly ED‐SCLC patients, but its effectiveness and clinical feasibility for elderly patients with treatment‐naïve ED‐SCLC have not been investigated. To our best knowledge, this is the first investigation to assess the efficacy and feasibility of atezolizumab, carboplatin, and etoposide for elderly patients with treatment‐naïve ED‐SCLC.

Our analysis showed that atezolizumab, carboplatin, and etoposide combination therapy is favorable against untreated ED‐SCLC in the elderly. The combination chemotherapy of carboplatin and etoposide has been demonstrated to be effective as a first‐line therapy for patients aged >70 years.^13^ A Japanese phase III study (JCOG 9702) comparing carboplatin and etoposide chemotherapy with split cisplatin and etoposide chemotherapy in patients aged ≥70 years with PS 0–2 and patients aged <70 years with PS 3 reported a 73% response rate in the carboplatin and etoposide arm and a median OS of 10.8 months in the group aged ≥70 years with PS 0–2.^13^ The phase III trial reported a median PFS of 5.2 months and a median OS of 10.6 months for the combination of carboplatin and etoposide arm.

Likewise, in a Japanese phase III trial comparing amrubicin monotherapy with carboplatin and etoposide chemotherapy in elderly (age ≥ 70 years) patients with untreated ED‐SCLC, the ORR in the carboplatin plus etoposide group was reported to be 60.0%, with a median PFS of 4.4 months and median OS of 11.3 months.^29^ The ORR of 80.5%, median PFS of 5.5 months, and median OS of 15.4 months in our elderly group is quite promising, compared with those achieved by conventional standard treatment of carboplatin and etoposide combination chemotherapy for elderly patients. Although the current analysis was a retrospective study, there was no significant difference in the response rate, median PFS, or median OS between the elderly and the non‐elderly groups. Moreover, age was not a significant prognostic factor for PFS or OS in both groups in the multivariate analysis (Figure S2). The ORR, median PFS, and median OS in our analysis compared favorably with the treatment effectiveness in the atezolizumab plus carboplatin and etoposide chemotherapy arm in the previous phase III IMpower133 study.^9^ Furthermore, in the IMpower133 study, 46% of the patients in the subgroup analysis were aged ≥65 years, and the PFS and OS in the atezolizumab group were 5.3 months and 12.5 months, respectively. The results of our study, which was conducted in a clinical practice setting and involved patients aged ≥70 years (considered as elderly), were in line with those of the IMpower133 study.

These discrepancies could be attributed to differences in patient backgrounds or other biases. However, our results are comparable to the median OS of 14.6 months in the Japanese subgroup analysis of the IMpower133 study,^30^ suggesting that Japanese patients may achieve a favorable OS. This may be because many patients in Japan undergo further treatment after disease progression, as is the case in our population. In our elderly patient population, 23 of 36 (63.8%) patients received second‐line therapy, and four patients received maintenance therapy. One patient was transferred to another doctor during treatment and thus could not be evaluated. The rate is lower in the IMpower 133 trial, wherein only 101 of the 201 (50.2%) patients in the atezolizumab plus carboplatin and etoposide combination chemotherapy arm received second‐line therapy.^9^ While previous studies of ICIs plus platinum and etoposide have focused on populations with a good PS (PS 0–1) and included only a small proportion of elderly patients,^9^, ^10^ our analysis included patients with PS 2–3 in both the overall and elderly populations. The results of good efficacy and tolerability suggest that combination chemotherapy with atezolizumab, carboplatin, and etoposide could be a standard treatment for elderly ED‐SCLC patients.

For further analysis, the elderly patients were divided into two cohorts by age: patients aged 70–74 years and those aged ≥75 years. As a result, no significant differences in the response rate, median PFS, and median OS were observed between the two groups (Table S1 and Figure S1). However, it is possible that the small number of patients (24 patients aged 70–74 years and 12 patients aged ≥75 years) prevented us from detecting significant differences. Future studies should include a larger sample size. It would also be clinically important to investigate the effectiveness and safety of this regimen in very older adult patients (i.e., those aged ≥80 years).

Univariate analysis of the data of the patients aged ≥70 years demonstrated that the PFS and OS did not significantly differ according to any of the clinical factors. These results suggest that combination chemotherapy of atezolizumab plus carboplatin and etoposide is universally effective in patients older than 70 years. However, further investigation could not be analyzed due to the small number of patients with a poor PS (≥2). Future studies on the efficacy of atezolizumab, carboplatin, and etoposide in patients with poor PS are warranted.

With respect to toxicity events, they were similar between the two cohorts in this study. However, the percentage of patients with decreased neutrophil count was higher in the current study than in the IMpower133 study,^9^ while the percentage of other adverse events was similar. Few immune‐related adverse events were observed, although this could be due to the small sample size of this study. Some patients developed hematological toxicity; of them, 2.7–61.1% had grade >3 toxicities. However, all hematological adverse events were manageable. Furthermore, non‐hematological adverse events were totally mild and manageable clinically. Pneumonitis occurred in two patients in the elderly group; both conditions resolved after steroid administration.

However, one patient in the elderly group had treatment‐related death due to bacterial infection. The patient received four cycles of atezolizumab, carboplatin, and etoposide combination chemotherapy and died of pneumonia and lung abscess after three cycles of atezolizumab maintenance therapy. It is likely that incidental infection developed when the patient achieved PR. In addition, the occurrence of toxicities was similar between the elderly and non‐elderly groups, although this was a comparison of a small population. These findings indicate that the adverse event signals of atezolizumab plus carboplatin and etoposide combination chemotherapy may be well tolerated by elderly SCLC patients.

Biomarkers such as tumor mutation burden and PD‐L1 protein expression lack accuracy for the identification of SCLC patients who will optimally benefit from atezolizumab plus carboplatin and etoposide combination chemotherapy.^19^ Similarly, there are currently no useful biomarkers for selecting alternative drug treatments. The limitations of PD‐L1 immunohistochemical analyses and the current lack of biomarkers for ICIs in SCLC underscore the need for additional research to further investigate potential biomarkers of ICIs in SCLC and their relevance to clinical outcomes.

There are some limitations in the current analysis. First, this was a retrospective study. In addition, it was an analysis with a small sample size; larger prospective trials are needed to verify the clinical usefulness of our findings. Second, the treating physician's decision was made to reduce, skip, or delay a treatment with anticancer agents. To reduce this bias to the greatest extent possible, all consecutive patients treated at the study sites were enrolled in the analysis, and their clinical records were thoroughly reviewed. Third, the administration of atezolizumab, carboplatin, and etoposide combination chemotherapy for first‐line treatment and the chemotherapeutic regimen for subsequent‐line treatments were determined by the attending physician based on the policy of each institution. These decisions could have introduced selection bias, which is an inherent limitation of retrospective studies. The possibility that this may have affected survival could not be ruled out. The treating physician's decision to reduce, skip, or delay a treatment with anticancer agents.

In conclusion, our real‐world data provide evidence that atezolizumab plus carboplatin and etoposide combination chemotherapy might be considered to be a feasible treatment strategy with favorable efficacy for treatment‐naïve elderly patients with SCLC. These findings may provide a new orientation in the pharmacological management of elderly patients with SCLC. In the future, it is necessary to conduct prospective studies on the effects of atezolizumab, carboplatin, and etoposide combination chemotherapy in elderly patients.

## AUTHOR CONTRIBUTIONS

All authors have read and approved the final manuscript. Conceptualization and methodology, A.S. and H.I.; formal analysis and data curation, H.I. and K.K.; Project administration, visualization, and writing—original draft preparation, A.S. and H.I.; Supervision, K.K. and H.K.; Investigation and resources, S.W., T.T., Y.N., H.M., Y.Y., T.K., Y.U., H.T., O.Y., A.M., H.T., and K.M.; writing—review and editing, all authors.

## CONFLICT OF INTEREST

The authors have declared that there are no competing interests.

## ETHICS STATEMENT

All procedures complied with the ethical standards of the institutional and/or national research committee and with the 1964 Helsinki Declaration and its later amendments or comparable ethical standards. The study design was approved by the Institutional Ethics Committee of International Medical Center, Saitama Medical University (No. 2021–113).

## INFORMED CONSENT

The requirement for written informed consent was waived by the ethics committee of Saitama Medical University because of the retrospective nature of the study.

## Supporting information


Figure S1

Figure S2

Table S1

Table S2

Table S3

## Data Availability

Expects Data Sharing
