# Supplementary figures and images for: Real‐world data of atezolizumab plus carboplatin and etoposide in elderly patients with extensive‐disease small‐cell lung cancer
Source: Cancer Med. 2022 Jun 14;12(1):73–83. doi: 10.1002/cam4.4938 (PMC9844637; doi:10.1002/cam4.4938)

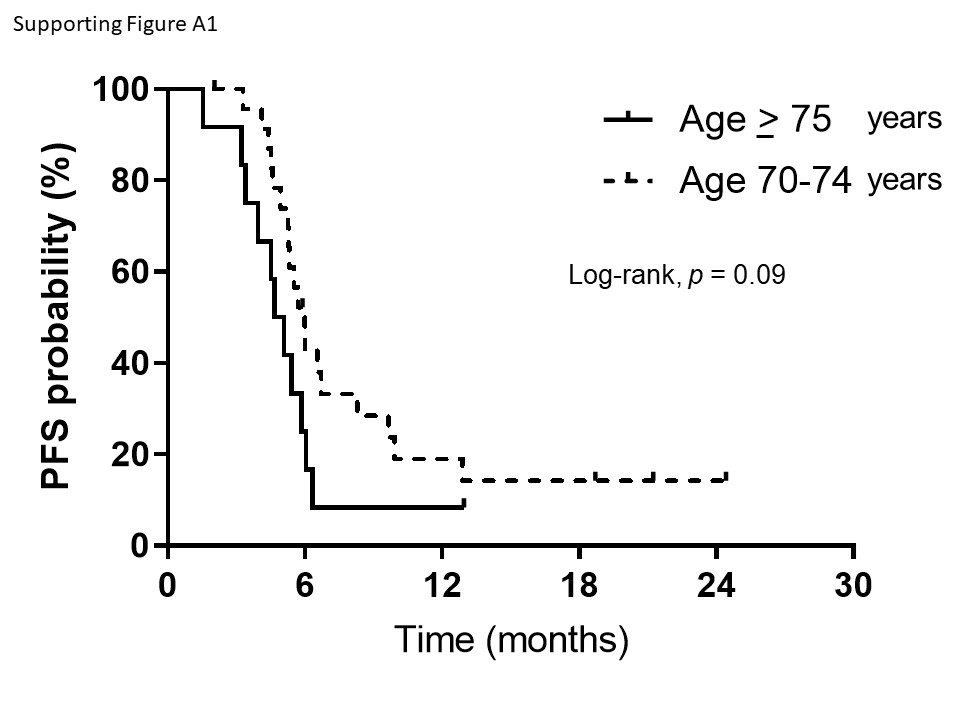

Supplement: Supplementary file 1 — Figure S1 [file CAM4-12-73-s004.JPG]

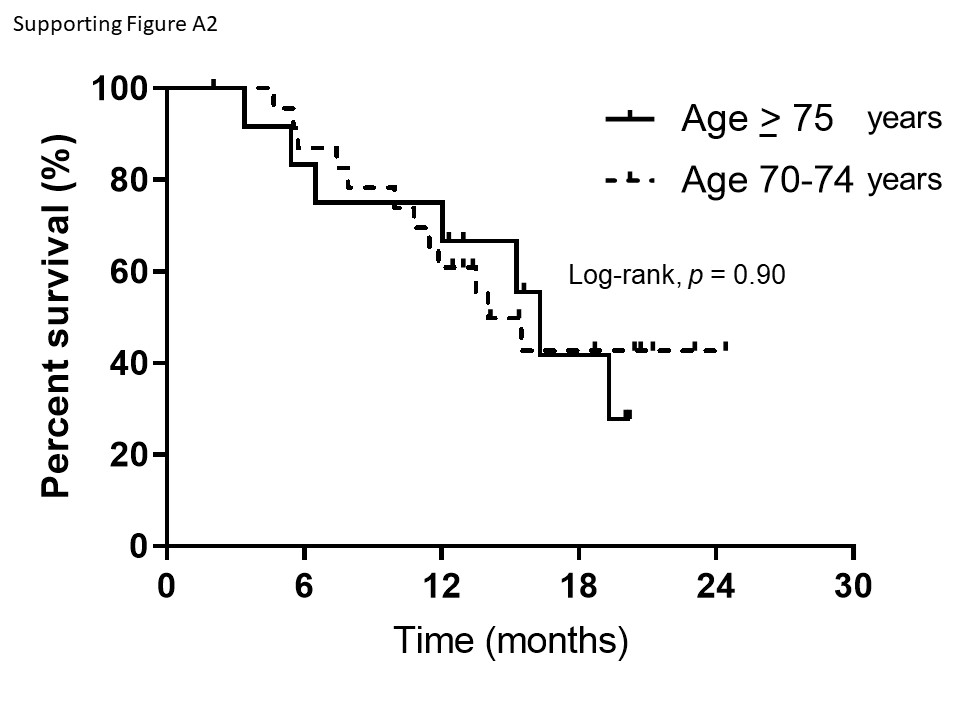

Supplement: Supplementary file 2 — Figure S2 [file CAM4-12-73-s001.JPG]
