# Supplementary material for: Real‐world data of atezolizumab plus carboplatin and etoposide in elderly patients with extensive‐disease small‐cell lung cancer
Source: Cancer Med. 2022 Jun 14;12(1):73–83. doi: 10.1002/cam4.4938 (PMC9844637; doi:10.1002/cam4.4938)
Supplement: Supplementary file 3 — Table S1 [file CAM4-12-73-s005.docx]

**Supporting Table S1.** Comparison of treatment response between patients aged 70–74 years and ≥75 years

|  | Age 70–74 years  (n=24) | Age ≥ 75 years  (n=12) | *p*-value |
| --- | --- | --- | --- |
| Response |  |  |  |
| CR | 1 | 1 |  |
| PR | 20 | 7 |  |
| SD | 2 | 2 |  |
| PD | 0 | 2 |  |
| NE | 1 | 0 |  |
| Response rate, % (95% CI) | 87.5 (68.1–96.4) | 66.6 (38.8–86.4) | 0.19 |
| Disease control rate, % (95% CI) | 95.8 (78.1–100.8) | 83.3 (53.9–96.5) | 0.25 |

CR Complete response; PR Partial response; SD Stable disease; PD Progressive disease; NE Not evaluated; CI, confidence interval
