# Supplementary material for: Real‐world data of atezolizumab plus carboplatin and etoposide in elderly patients with extensive‐disease small‐cell lung cancer
Source: Cancer Med. 2022 Jun 14;12(1):73–83. doi: 10.1002/cam4.4938 (PMC9844637; doi:10.1002/cam4.4938)
Supplement: Supplementary file 4 — Table S2 [file CAM4-12-73-s003.docx]

**Supporting Table S2.** Multivariate analyses of progression-free survival (PFS) and overall survival (OS) in all patient population

| Variables | Median PFS | Multivariate analysis | | | Median OS | Multivariate analysis | | |
| --- | --- | --- | --- | --- | --- | --- | --- | --- |
|  | (months) | HR | 95% CI | *p*-value | (months) | HR | 95% CI | *p*-value |
| Sex |  |  |  |  |  |  |  |  |
| Male / female | 5.5 / 5.2 | 1.13 | 0.48–2.86 | 0.77 | 15.9 / 18.3 | 1.17 | 0.45–3.35 | 0.74 |
| Age (years) |  |  |  |  |  |  |  |  |
| <70 / ≥ 70 | 4.9 / 5.5 | 1.54 | 0.88–2.69 | 0.12 | 15.9 / 15.4 | 1.57 | 0.81–3.05 | 0.17 |
| Intracranial metastases at initial treatment |  |  |  |  |  |  |  |  |
| Yes / no | 5.5 / 5.3 | 1.01 | 0.52–1.88 | 0.95 | 16.4 / 15.4 | 0.96 | 0.44–1.97 | 0.92 |
| Liver metastases at initial treatment |  |  |  |  |  |  |  |  |
| Yes / no | 5.4 / 5.3 | 1.71 | 0.83–3.32 | 0.13 | 9.9 / 16.4 | 2.07 | 0.89–4.41 | 0.08 |
| Bone metastases at initial treatment |  |  |  |  |  |  |  |  |
| Yes / no | 5.6 / 5.2 | 1.19 | 0.65–2.16 | 0.56 | 15.4 / 15.9 | 0.91 | 0.45–1.79 | 0.79 |
| Prior radiotherapy |  |  |  |  |  |  |  |  |
| Yes / no | 10.3 / 5.2 | 0.34 | 0.08–0.95 | 0.03 | 18.3 / 15.4 | 0.61 | 0.14–1.78 | 0.40 |

PFS progression-free survival; OS overall survival; HR hazard ratio; CI, confidence interval
