# Supplementary material for: Real‐world data of atezolizumab plus carboplatin and etoposide in elderly patients with extensive‐disease small‐cell lung cancer
Source: Cancer Med. 2022 Jun 14;12(1):73–83. doi: 10.1002/cam4.4938 (PMC9844637; doi:10.1002/cam4.4938)
Supplement: Supplementary file 5 — Table S3 [file CAM4-12-73-s002.docx]

**Supporting Table S3.** Univariate analyses of progression-free survival (PFS) and overall survival (OS) in the patients aged ≥70 years

| Variables | Median PFS | Univariate analysis | | | Median OS | Univariate analysis | | |
| --- | --- | --- | --- | --- | --- | --- | --- | --- |
|  | (months) | HR | 95% CI | *p*-value | (months) | HR | 95% CI | *p*-value |
| Sex |  |  |  |  |  |  |  |  |
| Male / female | 5.6 / 5.3 | 1.14 | 0.46–3.41 | 0.78 | 15.2 / NR | 1.66 | 0.47–10.49 | 0.46 |
| Age (years) |  |  |  |  |  |  |  |  |
| 70–74 / ≥ 75 | 5.9 / 4.8 | 0.53 | 0.25–1.16 | 0.11 | 14.0 / 16.3 | 0.94 | 0.39–2.63 | 0.90 |
| Intracranial metastases at initial treatment |  |  |  |  |  |  |  |  |
| Yes / no | 5.2 / 5.6 | 1.57 | 0.65–3.43 | 0.29 | 13.5 / 15.4 | 1.28 | 0.41–3.37 | 0.64 |
| Liver metastases at initial treatment |  |  |  |  |  |  |  |  |
| Yes / no | 5.5 / 5.5 | 1.40 | 0.61–2.99 | 0.40 | 13.5 / 16.3 | 1.43 | 0.45–3.77 | 0.50 |
| Bone metastases at initial treatment |  |  |  |  |  |  |  |  |
| Yes / no | 5.8 / 5.3 | 1.43 | 0.66–3.03 | 0.35 | 15.2 / 16.3 | 1.33 | 0.52–3.30 | 0.53 |
| Prior radiotherapy |  |  |  |  |  |  |  |  |
| Yes / no | 6.7 / 5.4 | 0.40 | 0.06–1.34 | 0.15 | NR / 15.2 | 0.33 | 0.01–1.62 | 0.20 |

PFS progression-free survival; OS overall survival; HR hazard ratio; CI, confidence interval; NR, not reported
